# Docking based 3D-QSAR studies applied at the BRAF inhibitors to understand the binding mechanism

**DOI:** 10.1186/1758-2946-4-S1-P46

**Published:** 2012-05-01

**Authors:** Uzma Mahmood, Zaheer ul-Haq

**Affiliations:** 1 Dr. Panjwani Center for Molecular Medicine and Drug Research, International Center for Chemical and Biological Sciences, University of Karachi, Karachi-75270, Pakistan

## 

B-RaF is a member of the RAF family of serine/threonine kinases involved in the regulation of cell growth, differentiation, and proliferation. It forms part of a conserved apoptosis signals through the RAS-RAF-MAPK pathway. As an important target of the cancer treatment B-Raf has more potential for researcher. The discovery of the ^V600E^B-RAF mutation [1] has elevated expectation for targeted therapy against human melanoma. In the current work the molecular modeling study were carried out very first time with 3D-QSAR studies [2] by correlating the docking protocol for three different datasets of B-Raf inhibitors. Based on the co-crystallized compound (PDB ID: 1UWJ), several alignment methods were utilized to derive reliable CoMFA and CoMSIA models. The best CoMFA model (q^2^ =0.753, r^2^ =0.807). With the same alignment protocol, a statistically reliable CoMSIA model # 14 was also derived (q^2^ = 0.962, r^2^ = 0.961). The actual predictive powers of both models were thoroughly validated with an external test set, which gave satisfactory predictive r^2^ values of 0.89 and 0.90, respectively. Contour maps from CoMFA and CoMSIA models, supporting the statistical results and revealed important structural features responsible for increasing biological activity within the active site and explained the correlation between biological activity and receptor-ligand interactions. These results can offer useful information for the design of new B-Raf inhibitor.
